# RETRACTION: Prevalence of Anxiety and its Risk Factors in Burn Patients: A Systematic Review and Meta‐Analysis

**DOI:** 10.1111/iwj.70270

**Published:** 2025-03-03

## Abstract

**RETRACTION:** MahdiabadiM. Z. , FarhadiB. , ShahroudiP. , ShahroudiP. , PourN. H. , HojjatiH. , NajafiM. , FarzanR. , and SalehiR. , “Prevalence of Anxiety and its Risk Factors in Burn Patients: A Systematic Review and Meta‐Analysis,” International Wound Journal 21, no. 2 (2024): e14705, 10.1111/iwj.14705.38353163 PMC10865278

The above article, published online on 14 February 2024, in Wiley Online Library (http://onlinelibrary.wiley.com/), has been retracted by agreement between the journal Editor in Chief, Professor Keith Harding; and John Wiley & Sons Ltd. A third party reported to the journal that they had found evidence of excessive self‐citations in the reference list of this article. The publisher confirmed multiple instances of redundant self‐citations. Upon further investigation, the publisher also concluded that this article was accepted solely on the basis of a compromised peer review process. In addition, the investigation found significant textual overlap between the methods and discussions sections of this article and another article by many of the same authors [1]. In view of the clear evidence of compromised peer review, the parties agreed that the paper must be retracted. The authors disagree with the retraction.

**Reference:**

[1] OtaghvarH. A. , FarzanR. , TamimiP. , GhaderiA. , NajafiM. , TohidianM. , et al., “Prevalence of Delirium and Its Related Factors in Burn Patients; a Systematic Review and Meta‐Analysis,” Archives of Academic Emergency Medicine 12, no. 1 (2024): e7, 10.22037/aaem.v12i1.2136.38162381 PMC10757577

**RETRACTION:** M. Z. Mahdiabadi , B. Farhadi , P. Shahroudi , P. Shahroudi , N. H. Pour , H. Hojjati , M. Najafi , R. Farzan , and R. Salehi , “Prevalence of Anxiety and its Risk Factors in Burn Patients: A Systematic Review and Meta‐Analysis,” International Wound Journal 21, no. 2 (2024): e14705, 10.1111/iwj.14705.38353163 PMC10865278

The above article, published online on 14 February 2024, in Wiley Online Library (http://onlinelibrary.wiley.com/), has been retracted by agreement between the journal Editor in Chief, Professor Keith Harding; and John Wiley & Sons Ltd. A third party reported to the journal that they had found evidence of excessive self‐citations in the reference list of this article. The publisher confirmed multiple instances of redundant self‐citations. Upon further investigation, the publisher also concluded that this article was accepted solely on the basis of a compromised peer review process. In addition, the investigation found significant textual overlap between the methods and discussions sections of this article and another article by many of the same authors [1]. In view of the clear evidence of compromised peer review, the parties agreed that the paper must be retracted. The authors disagree with the retraction.

## Reference

[1] H. A. Otaghvar , R. Farzan , P. Tamimi , A. Ghaderi , M. Najafi , M. Tohidian , et al., “Prevalence of Delirium and Its Related Factors in Burn Patients; a Systematic Review and Meta‐Analysis,” Archives of Academic Emergency Medicine 12, no. 1 (2024): e7, 10.22037/aaem.v12i1.2136.38162381 PMC10757577

